# Validation of *Diaporthe toxica* resistance markers in European *Lupinus angustifolius* germplasm and identification of novel resistance donors for marker-assisted selection

**DOI:** 10.1007/s13353-019-00521-y

**Published:** 2019-10-22

**Authors:** M. Książkiewicz, K. Wójcik, W. Irzykowski, W. Bielski, S. Rychel, J. Kaczmarek, P. Plewiński, E. Rudy, M. Jędryczka

**Affiliations:** Institute of Plant Genetics, Polish Academy of Sciences, Strzeszynska 34, 60-479 Poznań, Poland

**Keywords:** Narrow-leafed lupin, Molecular breeding, Pathogenic fungus, Phomopsis stem blight, *Phr1*, *PhtjR*

## Abstract

The fungus, *Diaporthe toxica*, anamorph *Phomopsis* sp., previously classified as *P. leptostromiformis*, is a plant endophyte and occasional pathogen, causing Phomopsis stem blight. This disease is damaging not only to lupins but also to the animals grazing on infected plants, due to the toxic secondary metabolites called phomopsins. The aim of this work was to validate markers for resistance to Phomopsis stem blight in narrow-leafed lupins and identify novel germplasm with increased levels of resistance to the disease. Plant inoculations were performed using ten isolates of *D. toxica*, originating from Australia and Poland. The European core collection of *L. angustifolius* was evaluated both in a controlled environment and with field experiments to classify the accessions based on their resistance to the disease. Simultaneously, the accessions were assayed with disease resistance markers to identify donors of hypothetical resistance alleles. We have found that the European lupin germplasm collection preserves wild and domesticated donors of at least two resistance genes to Phomopsis stem blight, including *Phr1* and *PhtjR*. Molecular markers PhtjM7, InDel2, and InDel10, tagging *PhtjR* gene, were applicable for marker-assisted selection targeting the European gene pool with an expected accuracy of 95%. None of diagnostic markers for the *Phr1* locus was found useful for European breeding programs; two existing markers Ph258M1 and Ph258M2 were unreliable, due to a high percentage of false-positive results (up to 58%) and a high recombination rate between markers (~ 30%).

**Electronic supplementary material:**

The online version of this article (10.1007/s13353-019-00521-y) contains supplementary material, which is available to authorized users.

## Introduction

The legume *Lupinus angustifolius* L. (narrow-leafed lupin) belongs to the genus *Lupinus* (tribe of Genisteae, family Fabaceae, subfamily Faboideae). It is well known as a source of protein for food and feed, as well as being a crop that contributes to the improvement of soil structure and fertility, increasing yields of succeeding crops (Peoples et al. 2009). Registered lupin cultivars are characterized by moderate grain yield with a high content of protein and oil, accompanied by low alkaloid content and limited fiber (Cernay et al. 2015; Lucas et al. 2015). Due to its relatively low chromosome number (2n = 40) and small genome size (2C = 1.89 pg), compared with other lupins (Naganowska et al. 2003), *L. angustifolius* became the species of choice for extensive molecular studies. Molecular research has been greatly facilitated by the development of bacterial artificial chromosome (BAC) libraries of the nuclear genomes for two *L. angustifolius* cultivars: Polish cv. Sonet (Kasprzak et al. 2006) and Australian cv. Tanjil (Gao et al. 2011). High-density linkage maps carrying thousands of markers, including gene-based sequence tagged sites (STS), were constructed and aligned to the draft genome sequence (Hane et al. 2017; Kamphuis et al. 2015; Nelson et al. 2006; Yang et al. 2013b). Exploitation of BAC resources for cytogenetic mapping resulted in the integration of all linkage groups with the corresponding chromosomes, as well as in the identification of several gene-rich regions (Książkiewicz et al. 2013, 2015; Leśniewska et al. 2011; Przysiecka et al. 2015; Wyrwa et al. 2016). The release of reference transcriptomes for wild and domesticated lupin accessions constituted a platform for targeting particular genes of interest (Kamphuis et al. 2015).

Despite its dynamic domestication history and high nutritional value, worldwide use of lupin for livestock feed has been hampered by high alkaloid content and the risk of lupinosis disease. The alkaloid issue has been almost entirely solved due to the discovery of three heritable factors decreasing alkaloid content (*iucundus*, *depressus*, and *esculentus*) (Hackbarth and Troll 1956). Incorporation of these alleles into breeding lines resulted in development of germplasm with a greatly reduced total alkaloid level, more than hundredfold lower than that of old cultivars (Kamel et al. 2016). However, lupinosis still remains a serious threat for animals grazing on lupin stubble (Cowley et al. 2014). The chemical factor causing lupinosis was revealed to be a phomopsin, a toxin produced by pathogenic fungi, *Diaporthe toxica* Will., Highet, Gams & Sivasith, anamorph *Phomopsis* sp. (Jago et al. 1982; Williamson et al. 1994). As the toxin is produced during the latent stage of stem infection of susceptible plants, exploitation of heritable resistance resources is a prerequisite of further lupin improvement. The fungus, formerly known as *Phomopsis leptostromiformis* (Kühn) Bubák, causes the lupin disease Phomopsis stem blight. Methods of screening for Phomopsis stem blight rely on observations of lesion coverage as percentage surface area on senescent stems (Cowling et al. 1987) or staining and microscopic examination of subcuticular coralloid hyphae structures of infected stems (Williamson et al. 1991). Additionally, a non-destructive glasshouse infection test has been developed, based on inoculation of lateral branches regenerating from the second main stem node, after topping the main stem above this node (Shankar et al. 2002). Over many years, *D. toxica* has been a major problem in Australia, where lupin was introduced as a winter crop. Australian breeders responded with the introduction of a moderately resistant wild population line from Morocco, CPI65211A, into a cross derivative of cv. Marri and P22872, which resulted in development of elite breeding line, 75A:258 (Cowling et al. 1987). The line is still used as a reference in phytopathological assays as its Phomopsis stem blight resistance has never been broken despite wide implementation of this genotype in Australian breeding programs (Shankar et al. 1996, 2002; Stefanova and Buirchell 2010; Yang et al. 2015, 2013a, 2002). In Europe, *D. toxica* seems to be a dormant pathogen as reports of the disease date from as early as 1880 in Germany and in 1892 in Denmark (on *L. angustifolius* and *L. luteus*) but it has never caused serious problems (Fischer 1893; Lind 1913). Although it has appeared from time to time in different countries in Europe, including Poland and Russia (Lewartowska et al. 1994; Marcinkowska 2007).

Australian lupin collection revealed extensive accession-related diversity in the severity of disease symptoms developed, from very susceptible lines (Unicrop, Uniharvest, Uniwhite), through susceptible (Chittick, Danja, Geebung), somewhat resistant (Merrit, Tanjil, and Wonga), to highly resistant (75A:258) (Yang et al. 2015; 2002). There are at least three different genetic sources of *D. toxica* resistance in *L. angustifolius*, all originating from an Australian collection. Reference germplasm resources for these genes are line 75A:258 (*Phr1* gene), cultivar Merrit (*Phr2*) and cv. Tanjil (*PhtjR*). With the use of the molecular fragment length polymorphism (MFLP) technique, markers linked to the putative *Phr1* resistance gene were designed, Ph258M1 and Ph258M2 (Yang et al. 2002). Next-generation sequencing of restriction site-associated DNA fragments was exploited to develop a set of single nucleotide polymorphism (SNP) markers linked to the *PhtjR* gene, namely PhtjM4, PhtjM5, and PhtjM7 (Yang et al. 2013a). Recently, a whole-genome resequencing approach was harnessed to develop a new set of low-cost markers tagging the *PhtjR* gene, including insertion/deletion PCR markers Markers InDel2 and InDel10, were found to be an effective diagnostic method on a broad range of Australian commercial cultivars (Yang et al. 2015).

Numerous undesired traits were eliminated during the history of narrow-leafed lupin domestication, including vernalization responsiveness, pod shattering, hard seed coat, bitter taste, and susceptibility to anthracnose. *L. angustifolius* breeding programs are most advanced in Australia; however, this process was largely based on two European genotypes (Borre and New Zealand Blue) and subsequently only occasionally improved with externally sourced germplasm (Berger et al. 2012). Only a small fraction of the available genetic and adaptive diversity was exploited during the domestication process of the species (Berger et al. 2013). Australian *D. toxica*-resistant cultivars cannot be exploited as highly transferable donors of resistance alleles due to significant genetic diversity constraints. The necessity for exploitation of wild populations and primitive forms to bypass the domestication bottleneck has emerged.

To address limitations of current breeding programs, we decided to leverage sources of *D. toxica* resistance from European *L. angustifolius* germplasm combining marker-assisted and classical approaches. Here, diagnostic procedures of Phomopsis stem blight resistance markers were optimized and the European core collection of *L. angustifolius* was assayed with these markers to identify hypothetical donors of resistance alleles. The resistance of selected narrow-leafed lupin lines was evaluated both in controlled environment tests and in field experiments to validate marker-trait associations as well as to identify novel germplasm with increased levels of Phomopsis stem blight resistance.

## Materials and methods

### Isolates of *Diaporthe toxica*

A collection of ten isolates of *D. toxica* was established (Table 1). Five isolates were collected from *L. luteus* plants, with four cultivated on fields located in the Wiatrowo Breeding Station of Poznan Plant Breeders and one was growing in the wild in Western Australia. The other five isolates were obtained from *L. angustifolius*, all of them were collected in Australia, mostly in Western Australia (4 sites) and one isolate was obtained from *L. angustifolius* collected in South Eastern Australia. The cultures of *D. toxica* (*Phomopsis*) were grown on PDA medium and the sporulation was induced under NUV light in growth chambers at 20 °C in the darkness.

**Table 1:** The isolates of *Diaporthe toxica* used in this study

No.	Isolate symbol	Host plant	Cultivar	Region	Location	Year
1	DTOX1	*L. luteus*	Juno	Greater Poland	Wiatrowo	2007
2	DTOX2		Mister			
3	DTOX3		Parys			
4	DTOX4		Juno			
5	WAC 8787	*L. angustifolius*	*wild plant*	Western Australia	Green Bushes	No data
6	DTOXA1				Perth	
7	DTOXA2				Perth	
8	WAC9513				Kojonup	
9	WAC8771				Wongan Hills	
10	WAC8782			South Eastern Australia	Wagga Wagga	

### Preliminary resistance survey

This experiment was conducted in 2007 in a phytotron (MYTRON Bio-und Solartechnik GmbH, Heiligenstadt) in controlled conditions (temperature regime 20 °C day/15 °C night, with 14-h day/10-h night photoperiod). Plants were grown in pots filled with sterilized soil substrate (Klassmann TS3 601 supplemented with the fertilizer PG Mix (Hartmann Ltd., PL). For each plant/pathogen combination, there were 10 plants treated in 3 replicates and 10 plants for mock inoculation. Lower parts of stems of 28-day plants were scarified by lancet (2 cm from root neck) and inoculated with 20 μL of conidia suspension of a given isolate of *D. toxica* (10^6^ conidia per ml). After inoculation, plants were grown in at least 80% relative humidity and a temperature regime of 22 °C day/19–20 °C night. High humidity (above 80%) was maintained using HADAR micro-sprinklers (NaanDanJain Irrigation, Naan, Israel). The test was performed using *L. luteus* Juno (Polish susceptible cultivar) and four *L. angustifolius* accessions: Sonet (Polish cultivar formerly used for BAC library development), breeding line 258 (Australian breeding line 75A:258 carrying putative resistance gene *Phr1*), Unicrop (Australian susceptible cultivar), and Tanjil (Australian cultivar carrying the putative resistance gene *PhtjR*). Four isolates were used for inoculation, Polish DTOX1 and DTOX4, and Australian DTOXA1 and DTOXA2 isolates (Table 1). The number of plants with visible Phomopsis stem blight symptoms was counted and expressed as percent of infected plants. Disease severity was scored 3, 7, 14, 21, and 30 days after inoculation and evaluated using the following scale:

- 0—resistance, no visible disease symptoms.
- 1—limited susceptibility, small light brown spots on the lower part of the main stem.
- 2—moderate susceptibility, medium-size brown lesions with isolated pycnidia.
- 3—high susceptibility, large brown lesions dispersed over all parts of the stem with pycnidia.
- 4—very high susceptibility, the whole stem covered by brown lesions with numerous pycnidia.

Statistical analysis of variance (ANOVA) was performed.

### Disease resistance evaluation in controlled conditions

Three experiments in a controlled environment (greenhouse) were performed in 2007 and 2008 (temperature regime 20 °C day/15 °C night). Seeds were sown in pots filled with sterilized soil. A total of 10 plants × 3 repeats for each line/pathogen combination and 10 plants for mock inoculation were assayed. The set of 26 *L. angustifolius* accessions analyzed in this survey contained 18 cultivars, 7 breeding lines, and one wild population (Online Resource 1). Inoculation was done using DTOX3 isolate. After inoculation, plants were grown with a relative humidity above 80% and a temperature regime of 22 °C day/19–20 °C night. The scoring of disease severity was performed 30 days after inoculation, using the same method as in the preliminary *D. toxica* resistance survey.

### Disease resistance assessment in field experiments

Field assays of Phomopsis stem blight resistance were performed in 2008–2009 from May to August at an area of 10 × 25 m, with 2 × 1 m of each plot. The cultivars of *L. angustifolius* (18) and *L. luteus* (7) delivered by Poznan Plant Breeders (breeding station Wiatrowo) and Plant Breeding Smolice Ltd., Co. (breeding station Przebędowo) were used (Online Resource 1). The experimental design comprised 3 replicates of inoculated plants and 1 control. Each replicate consisted of 3 field sections totaling 75 plants. Inoculation procedure, humidity control, and disease severity assessments were performed using the same methods as those used in the preliminary *D. toxica* resistance survey.

### PCR conditions

Primers were designed using Primer3Plus (Untergasser et al. 2007). Each PCR reaction was performed in a total volume of 20 μl in 96-well twin.tec PCR plates (Eppendorf, Hamburg, Germany) using 0.5 U Taq DNA Polymerase Recombinant (Invitrogen Thermo Fisher Scientific, Waltham, USA), 1× PCR buffer, 2 mM Mg^2+^, 0.25 mM dNTP, 0.25 μM of each primer, and 50 ng DNA template and deionized water. The amplification protocol included an initial denaturation at 94 °C for 4 min, followed by 35 cycles of annealing (45–62 °C for 30 s), elongation (72 °C for 40 s) and denaturation (94 °C for 30 s), and a final elongation step (72 °C for 6 min).

### Molecular marker detection—optimization and scoring

DNA was isolated from 3-week-old leaves using the DNeasy Plant Mini Kit (Qiagen, Hilden, Germany) according to the protocol. Two biological replicates were performed. The quality and concentration of isolated DNA were evaluated by two methods: agarose gel electrophoresis followed by ethidium bromide staining and spectrophotometer measurements (NanoDrop 2000; ThermoScientific, Waltham, USA). PCR primers (Table 2) were designed for the following markers tagging resistance genes: Ph258M1 and Ph258M2 for *Phr1* gene, and PhtjM7, InDel2 and InDel10 for *PhtjR* (Yang et al. 2015, 2013a, 2002).

**Table 2:** Primers used for optimization of *Diaporthe toxica* resistance markers

Marker	Primer sequences^a^	Target resistance gene	Enzyme and recognized sequence	Product lengths for resistant line (bp)	Product lengths for susceptible line (bp)
Ph258M1	TCCAGACTGACTATATTCTTAG CAGGCACATATATCTTTATACC	*Phr1*	–	303	254
Ph258M2_dCAPS	GGGAACAACAACAACAACTA GAACCATTGTAACTAAATCC	*Phr1*	*Mae*I CTAG	18, 185	206
PhtjM4_dCAPS1	TTCAACCAACGTGGGACTTAAATAGTTAA GTGGATACAACCTCACTGTC	*PhtjR*	*Hin*dII GTYRAC	89	25, 64
PhtjM4_dCAPS2	CAACCAACGTGGGACTTAAATATTTAA GTGGATACAACCTCACTGTC	*PhtjR*	*Aha*III TTTAAA	23, 64	87
PhtjM5_CAPS	GAATTCCATATGCAATGG CTTAATTGTTAATTTGTTATTTGC	*PhtjR*	*Cvi*JI RGCY	90	17, 73
PhtjM7_dCAPS1	CTTCAATTAGCTTGTCAGAAGACTTCCA CTAATTCAATGAGCTTCTCTT	*PhtjR*	*Nla*III CATG	27, 49	76
PhtjM7_dCAPS2	TTCAATTAGCTTGTCAGAAGACTCCAA CTAATTCAATGAGCTTCTCTT	*PhtjR*	*Sty*I CCWWGG	75	24, 51
InDel2	GATAAAGTATATCTAAATTATGTTTGC CTATATTTTGTATCAATTATAACAAATT	*PhtjR*	–	134	122
InDel10	GTTAAGTGGTAAATTGACTCATG GTTTTRCATTCTTGCAAAGATAAAATTAG	*PhtjR*	–	103	96

^a^The list contains primers developed in this study as well as those previously published (Yang et al. 2015, 2013a, 2002)

The optimization procedure involved PCR amplification using DNA isolated from reference *L. angustifolius* lines as a template (Tanjil, 75A:258, Unicrop) and amplicon sequencing. A range of annealing temperatures from 52 to 68 °C was tested. Length polymorphisms were visualized by 1% agarose gel electrophoresis (markers Ph258M1, InDel2, and InDel10), whereas nucleotide substitution polymorphisms were detected by the cleaved amplified polymorphic sequence (CAPS) (Konieczny and Ausubel 1993) and derived CAPS (dCAPS) (Neff et al. 1998) approaches (markers Ph258M2, PhtjM4, PhtjM5, and PhtjM7). Restriction sites were identified using dCAPS Finder 2.0 (Neff et al. 2002). Restriction products were separated by 1–3% agarose gel electrophoresis, with the agarose concentration adjusted according to the size of the expected digestion products.

The screening procedure was performed using Ph258M1, Ph258M2_dCAPS, PhtjM7_dCAPS2, InDel2, and InDel10 markers. The *L. angustifolius* germplasm collection used for genotyping consisted of 218 lines originating from 17 countries and differing by domestication status—which ranged from wild or primitive lines (76) through mutants (5) and cross derivatives/breeding lines (65) to cultivars (74) (Online Resource 1).

#### Validation of markers by disease resistance assay

The validation assay was done using 49 lines. The experiment was done in controlled environment in 2017 based on the results of *L. angustifolius* phenotyping against Phomopsis stem blight resistance and genotyping with Ph258M1, Ph258M2, PhtjM7, InDel2, and InDel10 markers. The inoculation of plants was done using two isolates showing the highest virulence against *L. angustifolius* (DTOXA2 and WAC8782). Inoculation method was similar to that used in greenhouse tests performed in 2007–2008. Disease scoring was done using the scale from 1 (immune, no symptoms) to 9 (fully susceptible with all symptoms typical to Phomopsis stem blight). Markers were validated by comparing the marker genotype with resistance phenotype by binary data similarity analysis. Taking into consideration the hypothesis that *D. toxica* resistance is conferred by dominant alleles (Shankar et al. 2002; Yang et al. 2002, 2013a), heterozygote and resistant homozygote marker scores were assigned as 1 and susceptible homozygote scores as 0. The same was done for phenotype observations—resistant and moderately resistant lines were marked as 1 and susceptible were marked as 0. Simple matching (Sokal and Michener 1958) and Rogers-Tanimoto (Rogers and Tanimoto 1960) coefficients were calculated using Binary Similarity Calculator http://www.minerazzi.com/tools/similarity/binary-similarity-calculator.php. Rogers-Tanimoto is a modification of the simple matching parameter that assigns double weight to mismatching variables, therefore emphasizing false-positive and false-negative scores.

## Results

### Germplasm resources resistant to *D. toxica* are preserved in the European *L. angustifolius* gene bank

The disease resistance survey under controlled conditions revealed that most of the analyzed *L. angustifolius* germplasm accessions were susceptible to *D. toxica* during the latent phase of the infection (Table 3, Online Resource 2). Susceptibility was demonstrated by the extensive colonization of stem tissues and formation of pycnidia. Susceptible lines were characterized both by high average disease severity scores and a high percentage of plants with visible disease symptoms. However, Australian cultivar Tanjil and German cultivar Arabella as well as Polish (W-226 and WTD-1406) and Australian (83A:476) breeding lines showed high levels of resistance to the pathogen, manifested by the lack of developed disease symptoms. Australian cultivar Myaille as well as Polish cultivar Bojar and German cultivar Boruta exhibited moderate resistance, with considerably delayed colonization of stem tissues and the development of only small light brown spots, limited to the lower part of the main stems.

**Table 3:** Results of *Diaporthe toxica* resistance assay in controlled environment (CE) and in field conditions

Species	Acc.	Line name	CE resistance	CE average score	CE average percentage	Field resistance	Field average score	Field average percentage
*L. luteus*	98072	Juno	**–**	**–**	**–**	S	4.0 ± 0.0	88.5 ± 4.9
	98153	Lord	**–**	**–**	**–**	S	4.0 ± 0.0	74.0 ± 9.9
	98145	Mister	**–**	**–**	**–**	S	3.0 ± 0.0	53.5 ± 13.4
	98136	Parys	**–**	**–**	**–**	S	4.0 ± 0.0	67.5 ± 7.8
	98154	Perkoz	**–**	**–**	**–**	S	3.5 ± 0.7	65.0 ± 14.1
	98150	Talar	**–**	**–**	**–**	S	3.5 ± 0.7	74.5 ± 31.8
	98148	Taper	**–**	**–**	**–**	S	3.5 ± 0.7	81.5 ± 0.7
*L. angustifolius*	–	Arabella	R	0.0 ± 0.0	0 ± 0	R	0.5 ± 0.7	12.5 ± 9.2
	96210	Baron	S	3.0 ± 0.0	78 ± 10	MR	1.0 ± 0.0	45.5 ± 24.7
	96225	Bojar	MR	0.3 ± 0.6	28 ± 48	R	0.0 ± 0.0	2.0 ± 2.8
	96211	Boruta	MR	0.7 ± 0.6	16 ± 15	MR	1.0 ± 0.0	16.5 ± 4.9
	96209	Elf	S	3.0 ± 0.0	78 ± 9	S	2.0 ± 0.0	18.5 ± 6.4
	96218	Graf	S	3.0 ± 0.0	88 ± 4	R	0.0 ± 0.0	0.0 ± 0.0
	96219	Kalif	S	2.0 ± 1.0	85 ± 4	MR	1.0 ± 0.0	24.5 ± 14.8
	95964	Karo	S	3.0 ± 0.0	83 ± 7	S	3.0 ± 0.0	73.5 ± 9.2
	95796	Mirela	S	2.3 ± 0.6	81 ± 12	S	3.0 ± 0.0	68.0 ± 4.2
	96185	Sonet	S	3.0 ± 0.0	81 ± 18	S	3.0 ± 0.0	91.5 ± 3.5
	96212	Zeus	S	3.0 ± 0.0	84 ± 10	S	3.0 ± 0.0	51.5 ± 2.1
	96233	83A:476	R	0.0 ± 0.0	0 ± 0	**–**	**–**	**–**
	96121	Emir	S	2.7 ± 0.6	90 ± 12	**–**	**–**	**–**
	96230	Mandelup	S	2.3 ± 0.6	88 ± 11	**–**	**–**	**–**
	96231	Myallie	MR	1.0 ± 1.7	22 ± 39	**–**	**–**	**–**
	96234	P27255	S	2.3 ± 0.6	76 ± 14	**–**	**–**	**–**
	96163	Polonez	S	3.0 ± 0.0	80 ± 8	**–**	**–**	**–**
	96214	Tanjil	R	0.0 ± 0.0	0 ± 0	**–**	**–**	**–**
	96102	Unicrop	S	4.0 ± 0.0	98 ± 4	**–**	**–**	**–**
	96222	W-197	S	2.7 ± 0.6	77 ± 3	**–**	**–**	**–**
	96223	W-211	S	3.0 ± 0.0	88 ± 7	**–**	**–**	**–**
	96224	W-226	R	0.0 ± 0.0	0 ± 0	**–**	**–**	**–**
	96196	W-89	S	2.3 ± 0.6	88 ± 0	**–**	**–**	**–**
	96183	Wersal	S	2.7 ± 0.6	78 ± 8	**–**	**–**	**–**
	96220	WTD-1305	S	3.0 ± 0.0	78 ± 5	**–**	**–**	**–**
	96221	WTD-1406	R	0.0 ± 0.0	0 ± 0	**–**	**–**	**–**

*acc.* accession, *CE* controlled environment. Resistance evaluation codes: *R* resistant, *MR* moderately resistant, *S* susceptible. Score: disease severity evaluation (0, resistant; 4, susceptible). Percentage: percentage of plants with visible disease symptoms developed

The field disease resistance survey included several *L. angustifolius* cultivars previously characterized in the controlled environment experiment as susceptible (Sonet, Karo, Zeus, Mirela, Graf, Elf, Kalif, Baron), moderately resistant (Bojar, Boruta), or resistant (Arabella), as well as seven *L. luteus* cultivars not yet evaluated for *D. toxica* susceptibility. Breeding lines were not assayed due to seed availability constraints. The results obtained for *L. angustifolius*-resistant and moderately resistant lines were consistent with those from the controlled environment experiment; however, some differences in the percentage of colonized plants or disease severity symptoms were observed, for example Bojar turned out to be less susceptible than Boruta. Moreover, significant differences in developed disease symptoms between field and controlled environment assessments were observed for *L. angustifolius* susceptible lines Kalif, Elf, and Graf, which had very low (Graf) or low (Elf and Kalif) levels of stem tissue colonization by *D. toxica*. All analyzed *L. luteus* cultivars exhibited high susceptibility to *D. toxica* (Table 3, Online Resource 2).

### Markers tagging *D. toxica* resistance genes are relevant to PCR-based genotyping

PCR products of markers Ph258M1, Ph258M2, PhtjM4, PhtjM5, PhtjM7, InDel2, and InDel10 (Yang et al. 2015, 2013a, 2002) were amplified using DNA isolated from reference lines (resistant and susceptible), sequenced, and compared with the target sequences. Amplification products with appropriate sequences were obtained for all analyzed markers. Markers Ph258M1, InDel2, and InDel10 were not sequenced as they were based on length differences of the amplified PCR products which could be directly visualized by simple agarose gel electrophoresis. Optimization was performed for certain markers: Ph258M2—anchored in a simple sequence repeat (SSR) locus; as well as PhtjM4, PhtjM5, and PhtjM7—tagging single nucleotide polymorphisms (SNPs). Ph258M2, PhtjM4, and PhtjM7 markers were converted to dCAPS markers, whereas PhtjM5 was converted to a CAPS marker (Table 2). Genotyping attempts with the use of reference lines and selected wild and domesticated accessions revealed that markers PhtjM4_dCAPS1, PhtjM4_dCAPS2, PhtjM5_CAPS, and PhtjM7_dCAPS1 had a very low rate of reproducibility resulting from weak PCR amplification and/or very low enzyme cleavage efficiency. On average, 4–5 replicates per sample had to be performed to obtain single score. Therefore, these four markers were discarded from further analysis. Marker sequences were aligned to the most recent genome assembly (Zhou et al. 2018). Markers PhtjM7, InDel2, and InDel10 and are localized in the same region of the chromosome CP023117.1 (LG-05) and are separated by 108,610 bp and 513 bp, respectively. Markers Ph258M1 and Ph258M2 are flanking the sequence of 269,913 bp in the chromosome CP023120.1 (LG-08). Alignment details are provided in Online Resource 3.

### At least two genetic sources of *D. toxica* resistance exist in the European *L. angustifolius* core collection

The European Lupin GenBank collection of 218 *L. angustifolius* accessions was screened with two markers tagging the *Phr1* gene (Ph258M1 and Ph258M2_dCAPS) and three markers linked to *PhtjR* gene (PhtjM7_dCAPS2, InDel2, and InDel10). Amplification products of expected sizes were obtained for 99.6% of samples. Additional alleles were very rare and occurred only in two lines: AN-80154a (marker Ph258M1) and W-226 (InDel2). These alleles were encoded as “R” homozygotes for marker validation procedure. Amplification consistently failed for Yorrel (PhtjM7_dCAPS2) and Badajoz-1 (InDel2). No single accession carrying R bands for all *Phr1* and *PhtjR* gene markers has been identified in the core collection. Therefore, it might be assumed that the analyzed germplasm array did not carry any line containing both *D. toxica* resistance genes in combination.

To enable comparison of marker genotypes with *D. toxica* resistance phenotype, a phytopathological assay in controlled environment was performed, involving 49 lines showing diverse combinations of marker scores. From 17 lines carrying both homozygous R alleles of Ph258M1 and Ph258M2 markers, only one (75A:258) was resistant in this experiment. From 13 lines analyzed, having at least two homozygous R alleles for PhtjM7, InDel2, and InDel10 markers, the resistance (or moderate resistance) was confirmed only for four accessions (Population B-551/79, Population 22695, Wonga and Tanjil). Moderate resistance was revealed also for Myallie and Boruta. These two lines carry S homozygous alleles for all tested markers but showed moderate resistance in one or two previous tests, respectively. On the contrary, the resistance of WTD-1406, W-226, and 83A:476, inferred from the initial controlled environment test, was not confirmed in this marker validation assay. The same phenomenon was observed for Arabella and Bojar which were revealed to be resistant in both previous tests. It should be noted that this test was highly selective, because some Australian lines reported in other studies (Shankar et al. 2002; Yang et al. 2002) as moderately resistant (Yorrel, Gunguru and Merrit) were revealed to be susceptible with an average disease scores from 3.6 to 3.8. Yet comparatively, reference lines 75A:258 and Wonga, carrying *Phr1* and *PhtjR* genes, were classified as resistant with scores 1.7 and 1.8, respectively.

### The set of PhtjM7, InDel2, and InDel10 markers is applicable for molecular selection of *PhtjR* gene

Marker genotype scores were compared with the results of this resistance survey as well as those published elsewhere. None of the markers displayed 100% consistency with *Phr1* and *PhtjR* genotypes inferred from phenotypic observations or published pedigree relationships among certain narrow-leafed lupin cultivars (Cowling 1999; Shankar et al. 2002; Stefanova and Buirchell 2010; Yang et al. 2002, 2015). Markers Ph258M1 and Ph258M2 generated higher number of false-positive results than markers PhtjM7, InDel2, and InDel10, namely 21 and 30 vs 6, 9, and 8. Low values of simple matching (0.48 and 0.38) and Rogers-Tanimoto (0.32 and 0.21) coefficients highlighted the negligible applicability of markers Ph258M1 and Ph258M2 for molecular breeding. Markers PhtjM7, InDel2, and InDel10 revealed to have higher values of both parameters, i.e., 0.63–0.68 for Rogers-Tanimoto and 0.77–0.81 for simple matching. All these three markers used together constitute relatively versatile selection tool, applicable for wide range of crosses (4% false-positive and 0% false-negative scores) (Table 4). The correlation between marker scores and phenotype observations enabled us to conclude that resistance of Population B-551/79 and Population 22695 lines is putatively conferred by the *PhtjR* gene (see Online Resource 4).

**Table 4:** Marker genotype scores for lines resistant to *Diaporthe toxica* evaluated in this study

Acc.	Name	Domestication status	2017 resistance	2017 score	Ph258M1	Ph258M2	PhtjM7	InDel2	InDel10
95744	Population B-551/79	WL	R	1.4 ± 0.7	S	R	R	R	R
26979	75A:258	BL	R	1.7 ± 1.3	R	R	S	S	S
96191	Wonga	CV	R	1.8 ± 1.3	S	S	R	R	R
96214	Tanjil	CV	R	2.2 ± 1.4	S	S	R	R	R
96231	Myallie	CV	MR	2.4 ± 0.8	S	S	S	S	S
95944	Population 22695	WL	MR	2.7 ± 0.7	S	R	S	R	R
96211	Boruta	CV	MR	2.7 ± 0.7	S	S	S	S	S
95964	Karo	CV	S	2.8 ± 0.8	S	R	S	S	S
96219	Kalif	CV	S	2.8 ± 1.6	S	S	S	S	S
95737	Population B-541/79	WL	S	2.9 ± 0.6	H	R	R	S	S
96110	Ignis	CV	S	2.9 ± 1.2	S	R	S	R	R
96170	R 83A,473	BL	S	2.9 ± 1.0	R	R	S	S	S
96233	83A:476	BL	S	2.9 ± 1.7	R	R	S	S	S
96235	Boregine	CV	S	2.9 ± 1.5	R	R	S	S	S
96195	Bolivio	CV	S	3.0 ± 1.4	R	R	S	S	S
96241	Vitabor	CV	S	3.0 ± 1.5	S	S	S	S	S
96185	Sonet	CV	S	3.1 ± 0.9	S	S	S	S	S
96212	Zeus	CV	S	3.1 ± 0.9	S	S	S	S	S
96223	W-211	BL	S	3.1 ± 1.6	S	S	S	S	S
–	Arabella	CV	S	3.1 ± 1.5	S	R	S	S	S
96230	Mandelup	CV	S	3.2 ± 1.4	R	R	S	S	S
96218	Graf	CV	S	3.3 ± 1.8	S	R	S	S	S
96240	Sonate	CV	S	3.3 ± 2.0	R	R	S	S	S
96102	Unicrop	CV	S	3.4 ± 1.7	S	S	S	S	S
96113	Frost	CV	S	3.4 ± 1.7	S	R	R	S	S
96224	W-226	BL	S	3.4 ± 1.6	S	S	R	R*	R
95726	Near Salamanca-b	WL	S	3.5 ± 0.9	S	R	R	R	H
96209	Elf	CV	S	3.5 ± 2.0	S	S	S	S	S
95843	Population 22660	WL	S	3.6 ± 1.7	R	R	S	S	S
95919	BRGC-10275	WL	S	3.6 ± 1.9	S	R	S	R	R
96161	Yorrel	CV	S	3.6 ± 1.5	R	R	S	S	S
96225	Bojar	CV	S	3.6 ± 1.2	S	S	S	S	S
95711	Badajoz 4	WL	S	3.8 ± 1.3	H	R	S	R	S
96162	Gunguru	CV	S	3.8 ± 2.1	R	R	S	S	S
96166	Merrit	CV	S	3.8 ± 1.7	R	R	S	S	S
96371	Population 1	WL	S	3.8 ± 1.7	S	R	S	R	R
96220	WTD-1305	BL	S	3.9 ± 1.4	R	R	H	S	S
95754	Population B-575/79	WL	S	4.0 ± 0.7	R	S	S	R	R
95840	AN-80154a	WL	S	4.0 ± 1.3	R*	R	R	S	S
96167	R 84A, 479	BL	S	4.0 ± 1.9	R	R	S	S	S
96221	WTD-1406	BL	S	4.0 ± 1.7	R	R	S	S	S
96183	Wersal	CV	S	4.2 ± 1.6	S	R	S	R	R
95842	Population 22661	WL	S	4.3 ± 1.7	R	R	S	S	S
96210	Baron	CV	S	4.3 ± 1.3	S	S	S	S	S
96222	W-197	BL	S	4.3 ± 2.0	S	S	S	S	S
96163	Polonez	CV	S	4.5 ± 1.7	S	S	S	S	S
95703	Hinojoso de Duero 3	WL	S	4.6 ± 1.4	H	H	S	R	R
95742	Population B-549/79b	WL	S	4.7 ± 1.7	R	R	S	S	S
96121	Emir	CV	S	5.2 ± 1.8	S	S	S	S	S
95796	Mirela	CV	S	–	S	R	S	S	S
96234	P27255	WL	S	–	R	R	S	S	S
96196	W-89	BL	S	–	S	S	S	S	S

*acc.* accession, *Ph* Phomopsis stem blight resistance evaluation. Domestication status codes: *CV* cultivar, *BL* breeding line or cross derivative, *WL* wild or primitive. Resistance evaluation codes: *R* resistant, *MR* moderately resistant, *S* susceptible. Marker genotype scores: *R* resistant allele, *H* heterozygote, *S* susceptible allele

*Additional allele present besides R allele

## Discussion

### *D. toxica* resistance genes in lupins

Resistance to Phomopsis stem blight in the narrow-leafed lupin is a complex trait and at least three genes are involved, originating from different germplasm resources, namely 75A:258, Merrit, and Tanjil. The highly resistant line 75A:258 was selected from a cross between cv. Marri and a wild line collected in Morocco (P22872) (Shankar et al. 2002). Moderately resistant cultivar Merrit was derived from a cross between cv. Illyarrie and a wild line from Spain (P22750) (Gladstones 1992). Resistant cultivar Wonga was derived from a cross between Gungurru and 75A54-5-8 (Stefanova and Buirchell 2010). The studies based on *F*_1_–*F*_3_ generations of crosses Unicrop × 75A:258 and Merrit × Unicrop revealed that both accessions carry independently segregating resistance alleles, named *Phr1* (75A:258) and *Phr2* (Merrit) (Shankar et al. 2002). *Phr1* was revealed to be fully dominant, whereas *Phr2* appeared to be incompletely dominant. Phenotyping studies based on crosses between Tanjil and other cultivars, including the susceptible Unicrop, revealed that Tanjil and Wonga possess a single-dominant *D. toxica* resistance gene which is different from *Phr1* and *Phr2*, this gene was named *PhtjR* (Yang et al. 2013a). Genetic analysis of *L. angustifolius* material from European breeding programs (Belarus, Russia, and Poland) with reference Australian lines Merrit and Gungurru also showed that resistance to *D. toxica* is determined by a single dominant gene, which was named *Rpl1* (Kuptsov et al. 2006). Several single dominant genes also control resistance against anthracnose in the narrow-leafed lupin germplasm, namely *Lanr1* in cv. Tanjil, *AnMan* in cv. Mandelup, and *LanrBo* in line Bo7212 (Fischer et al. 2015; Yang et al. 2004, 2008). In contrast, *D. toxica* resistance in another Old World lupin crop, white lupin (*Lupinus albus* L.), is under polygenic control as revealed by quantitative trait loci mapping in recombinant inbred line population derived from a cross between the susceptible Ukrainian cultivar Kiev Mutant and the resistant Ethiopian primitive P27174 accession (Cowley et al. 2014; Vipin et al. 2013).

### Tools for marker-assisted selection

At the time of their development, markers Ph258M1 and Ph258M2 were considered as useful for marker-assisted selection, with, however, a limited range of target crosses due to presence of false-positive scores in domesticated Australian germplasm, including cv. Merrit. This phenomenon resulted from the large distance between these markers and the *Phr1* gene, estimated to be 7.8 and 5.7 cM, respectively (Yang et al. 2002). Our study supported this observation providing evidence for both the occurrence of false-positive scores (accounting for 40–58% of analyzed lines) and the high rate of recombination between the markers (30% analyzed of lines, of which 63% were wild and 37% domesticated), see Online Resource 4. Better markers have not yet been developed because both existing *L. angustifolius* mapping populations are monomorphic for *Phr1* gene and could not be exploited to solve this issue (Boersma et al. 2005; Yang et al. 2013b). Harnessing of next-generation sequencing techniques based on whole-genome resequencing of reference resistant and susceptible lines may help to overcome this barrier in the near future and facilitate generation of high-quality, cost-effective markers, as it was the case for *L. angustifolius* anthracnose resistance (Yang et al. 2015).

Markers InDel2 and InDel10 originated from scaffold 84773 carrying the putative *PhtjR* resistance gene and were considered to be truly diagnostic since the marker genotypes were consistent with Phomopsis stem blight resistance phenotypes on all Australian cultivars analyzed (Yang et al. 2015). Our study showed that they are also diagnostic on a wide range of accessions from the European germplasm collection, including cultivars and breeding lines. We identified only three incidences of recombination events between InDel2 and InDel10 markers, which all occurred only in wild populations (Online Resource 4). Additionally, three heterozygote scores were found for InDel10, also only in wild populations. Marker PhtjM7, located roughly 1 cM from the target *PhtjR* gene, was originally recommended as applicable for marker-assisted selection in narrow-leafed lupin breeding due to the lack of recombination between the marker and the gene in the set of 26 Australian cultivars; expected genotyping accuracy was estimated to be approximately 99% (Yang et al. 2013a). However, we identified the presence of recombination between PhtjM7 and InDel2 or InDel10 markers in as many as 30 lines, including 22 wild and 8 domesticated lines. Nevertheless, this marker had slightly higher genotype to phenotype similarity coefficients than InDel2 or InDel10 in the validation assay. All three markers used together provided ~ 95% confidence on selection of the desirable *PhtjR* allele.

The most reliable markers for genotype selection are those anchored in functional mutations of genes conferring particular traits, as was established for *L. angustifolius* vernalization responsiveness locus *Ku* and corresponding large deletion in the promoter region of *Flowering locus T* homolog, *LanFTc1* gene (Nelson et al. 2017). Nevertheless, deciphering the molecular background underlying the *Ku* locus took more than decade of extensive research involving various techniques, from BAC library screening via DNA hybridization, restriction site-associated physical and linkage mapping, fluorescent in situ hybridization of DNA probes in metaphase chromosomes, Sanger, 454 and Hi-seq sequencing to gene expression profiling (Książkiewicz et al. 2016; Nelson et al. 2017, 2006). Although a similar BAC library approach was applied to *L. angustifolius* Phomopsis stem blight resistance markers PhtjM2 (linked with *PhtjR*) and Ph258M2 (*Phr1*), it did not yield resistance gene identification or the development of tightly linked diagnostic markers (Książkiewicz et al. 2013, 2015). Those studies showed convincingly that MFLP-derived probes are not suitable for positional cloning of particular genes because they hybridize to numerous loci dispersed in the genome, localized both in repetitive and gene-rich regions.

The falling price of sequencing and the increase in the size of sequence databases has reduced the cost of obtaining useful sequence information for analysis (Muir et al. 2016). Accelerating progress in next-generation sequencing technology and annotation has considerably shortened the time and money required for identifying genes controlling agronomic traits, as was demonstrated for *LpPg1* stem rust resistance locus in perennial ryegrass (*Lolium perenne*) where the NBS-LRR gene was revealed through massive analysis of cDNA ends (MACE) (Bojahr et al. 2016). The MACE approach has also been implemented in *L. angustifolius* applied research, aimed at providing ready-to-use technology for gene-based monitoring of key agronomic traits including, among others, resistance to pathogenic fungi (Książkiewicz et al. unpublished).

Both identified resistant wild lines (Population B-551/79 and Population 22695) originate from Spain. The geographical localization of these putative resistance donors in warm Mediterranean climates coincides with the ecological niche of the pathogen. Thus, in controlled conditions, the maximum infection efficiency was observed after a dew period of 48–72 h with temperatures within a range of 15–25 °C with 20 °C being the optimum (Williamson and Sivasithamparam 1994). In Australian field conditions, infections and subsequent lupinosis appearance were determined to be correlated with rainfall patterns (Cowling and Wood 1989; Petterson and Wood 1986). Phomopsis stem blight has been considered by European lupin breeders as an unimportant disease, constituting a threat to white lupin only in wet years (Święcicki and Święcicki 1995). However, the advance of global warming may expand the *D. toxica* climatic optimum in to the north, reaching major European areas of lupin cultivation and forcing adaptation of breeding strategies to develop crop cultivars adapted to new threats. Unfortunately, much of the natural genetic diversity in narrow-leafed lupin has been lost during the domestication process (Berger et al. 2012). Incorporation of alleles from wild germplasm to widen the genetic diversity of the domesticated pool is currently emerging as an important need of the narrow-leafed lupin breeding community (Berger et al. 2013). This process may be facilitated by using *PhtjR* molecular markers validated in this study for European lupin germplasm.

## Conclusions

1. The European lupin germplasm collection preserves wild and domesticated donors of at least two Phomopsis stem blight resistance genes, *Phr1* and *PhtjR*.
2. The set of molecular markers PhtjM7, InDel2, and InDel10, tagging the *PhtjR* gene, can be used in marker-assisted selection targeting the European gene pool with an expected confidence about 95%.
3. By now, no reliable diagnostic marker for the *Phr1* applicable for European breeding programs has been found; the two existing markers showed high percentage of false-positive results and high recombination rates between markers.

## Electronic supplementary material

Online Resource 1 List of *Lupinus* spp. accessions used for Phomopsis stem blight resistance profiling and Ph258M1, Ph258M2, PhtjM7, InDel2 and InDel10 marker genotyping. (XLS 79.5 kb)

Online Resource 2 Phomopsis stem blight resistance evaluation of *Lupinus angustifolius* in field and controlled environment (CE) conditions. (XLS 51.5 kb)

Online Resource 3 Results of alignment of Ph258M1, Ph258M2, PhtjM7, InDel2 and InDel10 marker sequences to the *Lupinus angustifolius* genome assembly. (XLSX 11.7 kb)

Online Resource 4 Results of *Lupinus angustifolius* germplasm genotyping with Ph258M1, Ph258M2, PhtjM7, InDel2 and InDel10 markers tagging Phomopsis stem blight resistance genes. (XLS 63.0 kb)
